# Towards Double Defense Network Security Based on Multi-Identifier Network Architecture

**DOI:** 10.3390/s22030747

**Published:** 2022-01-19

**Authors:** Yunmin Wang, Abla Smahi, Huayu Zhang, Hui Li

**Affiliations:** 1 School of Electronic and Computer Engineering, Peking University, Shenzhen 518055, China; wangyunmin@pku.edu.cn (Y.W.); smahi_abla@pku.edu.cn (A.S.); 2 Purple Mountain Laboratories, Nanjing 211111, China; 1301111205@sz.pku.edu.cn; 3 Peking University Lab of China Environment for Network Innovation, Peking University, Shenzhen 518055, China

**Keywords:** network security, double defense, zero trust, situation awareness, immunology

## Abstract

Recently, more and more mobile devices have been connected to the Internet. The Internet environment is complicated, and network security incidents emerge endlessly. Traditional *blocking and killing* passive defense measures cannot fundamentally meet the network security requirements. Inspired by the heuristic establishment of multiple lines of defense in immunology, we designed and prototyped a Double Defense strategy with Endogenous Safety and Security (DDESS) based on multi-identifier network (MIN) architecture. DDESS adopts the idea of a zero-trust network, with identity authentication as the core for access control, which solves security problems of traditional IP networks. In addition, DDESS achieves individual static security defense through encryption and decryption, consortium blockchain, trusted computing whitelist, and remote attestation strategies. At the same time, with the dynamic collection of data traffic and access logs, as well as the understanding and prediction of the situation, DDESS can realize the situation awareness of network security and the cultivation of immune vaccines against unknown network attacks, thus achieving the active herd defense of network security.

## 1. Introduction

With the development of the Internet and its deep integration with human social life, more and more mobile devices are connected. They are heterogeneous and ubiquitous, and put forward higher requirements for network security. The predominant *best-effort* design of TCP/IP network architecture focuses on the end-to-end communication between non-commercially and mutually trusted users. The network data transmission is entirely transparent, and the service and the bearer are separated. However, it instead hands over the security control to the users. This helps to improve the network availability and flexibility but at the cost of network security [1]. The Internet appears to be incapable of responding to users’ demands for security in obtaining massive content. In addition, the current internet IP addresses have positioning, identity, and forwarding functions that pose many challenges for supporting quality of service (QoS), especially for the Internet of things (IoT) [2], Internet of Vehicles (IoV) [3], and the authenticity and credibility of the fusion of the virtual and real world in the metaverse [4].

**Figure 1:**
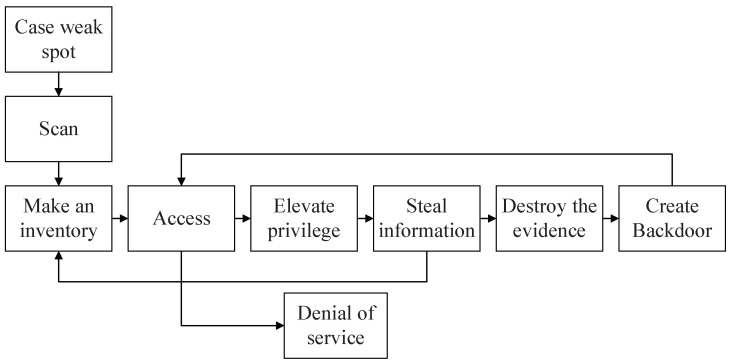
Network attack procedures.

Currently, network security protection is mainly based on IP networks’ characteristics and adopts passive defense measures with *blocking and killing* methods, such as firewalls, authentication technology, access control, vulnerability scanning, disaster recovery, and honeypot technology. The defense capabilities of these measures can be passive or static, depending on predetermined settings before accessing the system and updating the preset defense library during use. Therefore, they can only detect and defend against a number of predefined network security attacks. Moreover, although these methods require higher user permissions and privileges, they can be easily controlled and exploited by attackers [5]. It is worth mentioning that the network security vulnerabilities constantly emerge while the attack methods are persistently refreshed. As demonstrated in Figure 1, attackers usually scan the weakest link in security defense to launch network attacks. Traditional defense measures focus on improving the protection capabilities against attacks rather than identifying, tracking, and investigating the responsibility of the attackers. They passively receive every intrusion attack, which is difficult to detect, identify, and respond to emerging attack methods, and it is challenging to solve network security problems fundamentally.

**Figure 2:**
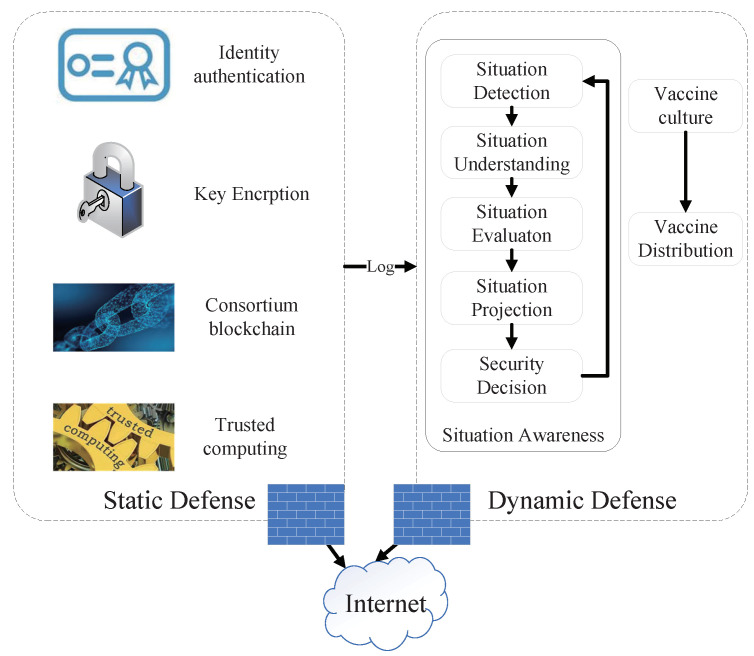
Double defense strategy with endogenous safety and security (DDESS).

While paying attention to network security, we found much inspiration from the biological immune system. The biological immune system has the advantages of feature extraction, distributed detection, self-tolerance, self-adaptation, robustness, and the capabilities of pattern recognition, learning, and memory [6]. It forms individual immunity to viral infections through its physical barrier, innate immune system, and adaptive immune system, and achieves the public herd immunity through collaboration between heterogeneous nodes and the cultivation and injection of vaccines [7]. Inspired by the multiple defense lines in immunology [8], this paper proposes a double defense strategy with endogenous safety and security (DDESS) as shown in Figure 2 based on the multi-identifier network (MIN) architecture [9]. We adopt identity authentication as the core access control method to solve traditional IP network security problems, and implement static network security defense through key encryption technology, blockchain technology, and trusted computing whitelist strategy. At the same time, through the dynamic collection of data traffic and access logs, DDESS can find the network vulnerabilities in the existing system, understand and predict the situation, realize the situation awareness of network attacks, and cultivate the immune vaccine of unknown network attacks, to complete the active dynamic defense of network security and the balance of network availability and security.

In summary, this paper makes the following contributions:

- This paper discusses the problem of the network’s bottleneck resulting from the traditional IP-network carrying capacity and the poor network security. In this regard, we inspire our proposed system from the idea of zero-trust network [10]. In doing so, based on MIN architecture [9,11], we built a security strategy with an identity identifier as the core of network transmission. This strategy is built based on identity identifiers rather than network locations. Only after user authentication, authorization, and account verification can the user and the application communicate.
- Signature of network transmission packets protects data from theft. A variety of the data related to the identifiers is stored in the consortium blockchain of the MIN to ensure the identifier data’s non-repudiation, tampering-resistance, and traceability. The independently developed voting consensus algorithm Proof of Vote (PoV) [12] improves the throughput of the system. At the same time, the encryption key and data are stored in the trusted computing modules (TCM) to ensure that they are not read or disclosed [13], and protected by remote authentication [14]. Real-time monitoring of the trusted whitelist of accessing applications can promptly detect and respond to attacks.
- The network traffic and application access logs are collected and analyzed for further situation awareness. DDESS classifies and extracts appropriate attack information, including attack behavior and mode, and makes use of the convolutional neural networks (CNN) to evaluate the network security attack index of attack information. In addition, DDESS predicts the network security of the existing network system and provides the corresponding solutions. DDESS simulates network behaviors in the sandbox. It then analyzes these simulation results, cultivates a security immune vaccine, enriches the network attack behavior database, and prevents unknown network attack behaviors.

The remainder of this paper is organized as follows: Section 2 reviews the related work of image anomaly detection. A detailed description of our proposed method is given in Section 3. In Section 4, we present experimental setups and comparisons. Section 5 carries out the corresponding experiments and gives out the simulations results. Section 6 concludes the paper and points out some of our future research.

## 2. Related Work

The traditional static defense of network security is divided into three types: reinforcement protection of the system, intrusion detection, and network deception [15].

Firewall (including packet filtering, proxy type, state inspection, in-depth inspection, web application) [16], encryption and decryption, data authentication [17], and access control [18] focus on protecting information and enhancing the security of the network system itself. They play a protective role in ensuring the normal access channels of the network system, authenticating legitimate user identities and rights management, and the security of confidential data and information.

Intrusion detection [19], vulnerability detection [20], traffic analysis [21], log auditing [22], and other attack methods for known characteristic information make use of methods such as characteristic scanning, pattern matching, and comprehensive data analysis to conduct dynamic monitoring, linkage alarms, and emergency response to prevent or eliminate attack threats.

Honeypot technology [23] deploys some hosts and network services as decoys to induce attackers to carry out attacks, thereby capturing and analyzing the attack behaviors, understanding the tools and methods used by the supplier, and speculating the intention and motivation of the attack, thereby enhancing its security protection capabilities [24]. However, it is difficult to deploy a honeypot environment that is not readily perceivable by intruders [25].

The above static defense methods can better defend against attacks with known characteristics and fixed patterns. However, it cannot defend against attacks based on unknown vulnerabilities backdoors, complicated and changeable multimode joint attacks, and attacks from within the network. With the continuous improvement of the automation and intelligence of countermeasures, the construction and strengthening of the network security defense system alone can no longer meet the actual needs of network security defense. Dynamic defense technology has gradually attracted widespread attention and is considered a revolutionary technology that changes the asymmetry of network security, such as Moving Target Defense (MTD) [26], Cyberspace Mimic Defense (CMD) [27], and Cyber Deception (CD) [28].

Let us dive into a specific scenario: vulnerabilities have constantly been discovered in the Internet of Vehicles (IoV), and the security problems of Vehicle to Everything (V2X) interactive communication are gradually emerging. The Internet of Vehicles requires real-time and timely resolution of network security issues. Therefore, many machine learning-based solutions in V2X scenarios have been proposed to provide dynamic defenses and address these problems. The surveillance of physical layer security (PLS) was explored in the field of connected vehicles [29]. A delimitated anti jammer scheme based on machine learning [30] secures the network and alleviates the traffic congestion simultaneously; in the meantime, it can reduce the computing delay.

Network dynamic defense is an innovative network defense technology system gradually developed to deal with the increasingly severe cyberspace security situation, which makes it possible to break the long-standing impregnable asymmetry and will balance the difficulty of network attack and defense in the future.

In this paper, we present a network defense strategy that integrates static and dynamic defenses. It adopts the ideas of the zero-trust network, and employs identity authentication, blockchain technology, and trusted computing technology, with situation awareness and dynamic immune functions.

## 3. Static Defense

We adopt the idea of a zero-trust network and build a multi-identifier network system with identity as the core, supplemented by data signature, blockchain technology, and trusted computing technology to realize the static defense of network security to improve the autoimmunity of network individuals.

### 3.1. Multi-Identifier Network System with Identity as the Core

The network identifier is the data carried in the network packet for addressing and forwarding by the intermediate router. In the traditional IP network, the target IP address of a network packet is its network identifier, forming a thin waist hourglass structure with IP as the core in the network layer, which identifies two network nodes for end-to-end communication. This network architecture is no longer suitable for the network communication requirements of the existing network to obtain content and services. At the same time, since the original design purpose of the IP network is to communicate between non-commercial users who trust each other, the network transmission is entirely transparent to the intermediate router, which makes data theft and monitoring easy. The IP network lacks top-level design and usually adopts a passive patching method for network security, making it increasingly challenging to implement [31].

**Figure 3:**
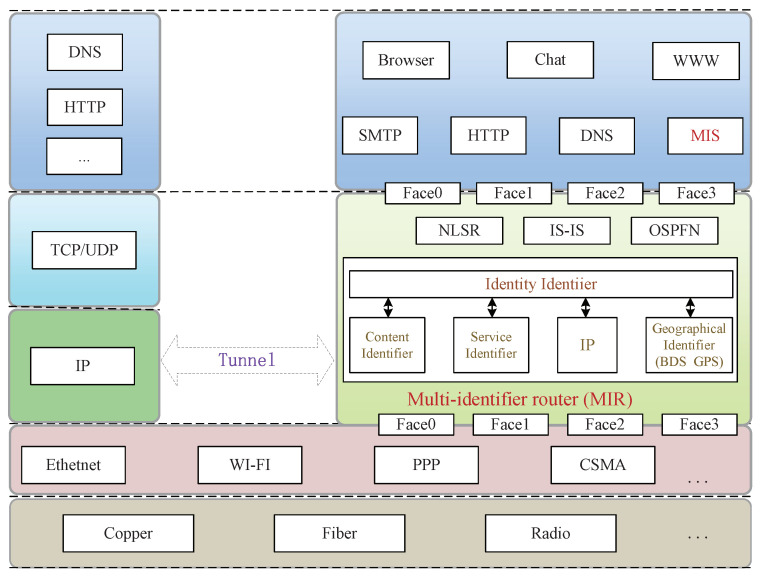
Multi-identifier network architecture.

**Figure 4:**
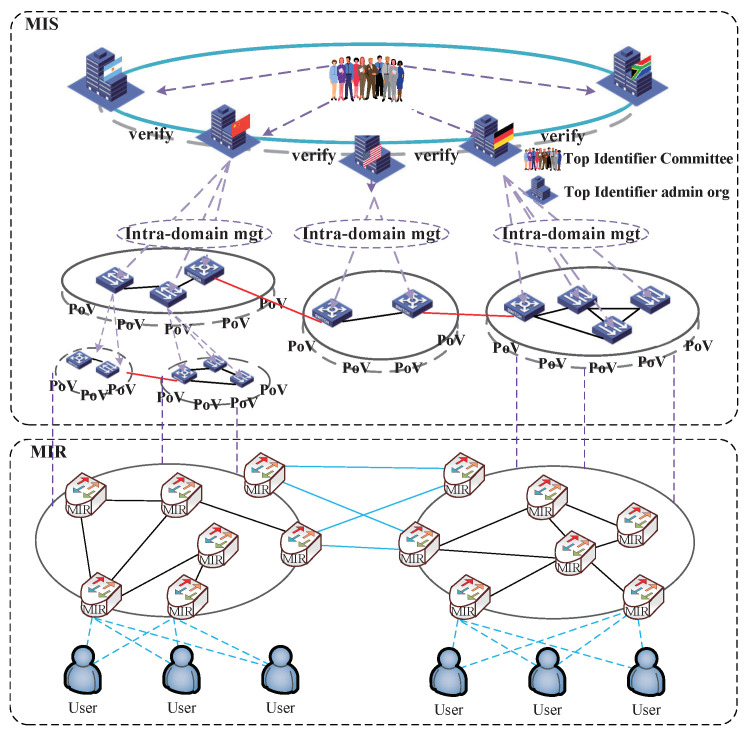
Separation of multi-identifier network management plane and data plane.

The multi-identifier network (MIN) system [11] takes identity as the core, and supports the coexistence of multiple network identifiers such as identity, content [32], service, space, space, and location information, and IP, to solve the depletion of IP addresses and the security problems existing in IP networks, as shown in Figure 3. The multi-identifier network is divided into a management plane (multi-identifier system, MIS) and data plane (multi-identifier routers, MIR), which supports simultaneous transmission of multiple network identifiers in the network, as depicted in Figure 4. MIS supports the management and resolution of network identifiers by multiple parties equally, and is responsible for the affairs related to identifiers combined with offline. Its main functions include identifier registration, identifier query, identifier generation, and identifier query. The multi-identifier router (MIR) is mainly responsible for the addressing, forwarding, and mutual translation functions of multiple identifier network packets. It supports the push transmission mode of identity and IP identifiers and the pull transmission mode of content identifier and service identifier.

Identity is not limited to users but also includes the unique identification of network communication physical entities (from now on referred to as users) such as devices, interfaces, applications, and business systems in the digital network world. It is the equivalent of physical network entities in the digital network world. The identity of a physical entity is unique in the digital network world, which is different from a username. A physical entity can have different usernames in different systems.

The identity identifier is the core network identifier in the MIN, and all multi-identifier routers need to support routing and forwarding of the identity identifier. Other types of network identifiers will be associated with a particular identity identifier. In an unsupported network domain, it can go back to the identity identifier for data forwarding. The hash value of the real identity information of the network communication entity (such as ID number, fingerprint, face, voiceprint, iris, and other biometric information, password) is included in the signature field in the data packet. It carries the sender’s accurate identity signature information and time stamps, realizes dynamic access control based on identity, establishes a unified digital identity identifier and life cycle management for users and other physical entities participating in network communications, and saves them in the consortium blockchain of the MIS system.

After the communication entity is registered in the MIS, a unique identity is formed, corresponding permissions are assigned according to its role identification, and fine-grained permission management is carried out by monitoring its access behavior to realize the management and control of dynamic permissions. The principle of the least authority is implemented for communication entities, and their authority is allocated reasonably to ensure that each communication entity (including applications, services, users) can only access the required information or resources. When it is necessary to access sensitive resources, the identity recognition module will call other real identity information saved in the MIS for secondary verification, such as SMS verification code, dynamic password, face verification, to form access control and dynamic threat identification, privilege confirmation, alarm, and blocking at the regional boundary. The information transferred by users in the system will be recorded. The data package is highly bound with the user identity information to realize the traceability of data and behaviors.

### 3.2. Encryption and Blockchain Protection

Identity authentication is based on Elliptic Curve Cryptography (ECC), rather than RSA. In addition to the registration phase, the subsequent authentication phase does not require the participation of a trusted third party, which ensures communication security and reduces the overhead of computing and communication. Data transmitted in the network will be protected by hash and asymmetric encryption at the packet level, rather than at the channel level, to prevent sensitive information from being eavesdropped, intercepted, or tampered with.

The nodes in the MIS consortium chain are divided into committee nodes, accounting nodes, and ordinary user nodes. The committee nodes are elected by members who volunteer to maintain the consortium chain and have equal voting rights jointly. The bookkeeping node is a node with the privilege to produce blocks voted by the committee nodes.

During user registration, the user will generate his public and private key pair, package the public key and the real identity information signed by the private key, and submit it to any blockchain node in the MIS system. The node will check the format of the registration request and find whether the user information already exists in the local database to avoid repeated registration. After that, this node will perform primary verification of the content of the registration information, package it into blockchain transaction information and submit it to the accounting node in the MIS consortium blockchain, and store the transaction information in the transaction pool.

**Figure 5:**
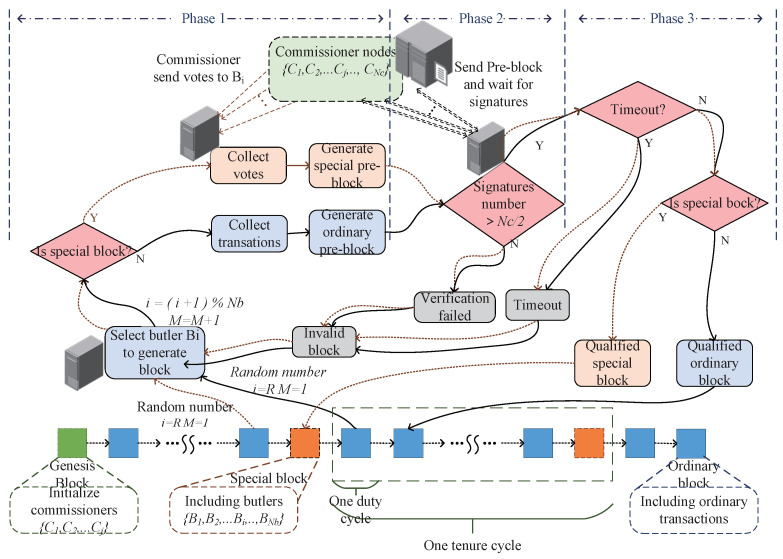
PoV consensus and block generation procedure.

We adopted the PoV consensus algorithm [33] to accelerate the process of consortium blockchain consensus, as depicted in Figure 5. At the beginning of the blockchain consensus, the accounting node will take out some transaction information from the transaction pool to generate a pre-block, and send it to the committee node in the consortium chain to request a signature. The committee node verifies the pre-block header and the content of each transaction, and sends it back to the accounting node after signing. If the billing node receives more than half of the signatures of the committee nodes within a given time threshold, it will store the signature information in the block header and set the timestamp to write this pre-block to the master of the consortium chain, and the pre-block becomes an official block. Otherwise, the pre-block will be deleted; the transaction will be taken out from the transaction pool again, and the pre-block will be generated and sent to all committee nodes for verification and signature.

When a user performs network data transmission, the user’s signature information will be included in the data packet to record the user’s access path in the multi-identifier network and realize the traceability of network security. When abnormal access is detected, the registered identity information reserved by the user will be retrieved from the consortium chain database, and the user will be warned or prohibited.

### 3.3. Trusted Computing Whitelist

Trustworthiness is the expectation that an entity can acquire the expected effect when realizing a given goal. It should be salable, adaptive, and auto-configurable, especially for the ubiquitous computing scenarios, such as Internet of Things and Internet of Vehicles [34]. We adopted the active immune trusted computing module (TCM) independently developed by China [13] in the client host hardware to save the encryption keys used in the consensus process of the blockchain nodes.

The private key is stored in the TCM chip and cannot be leaked and read. The public key can be used everywhere to identify the node identity and for signature verification. At the same time, all kinds of data generated by the node (such as blocks, transactions) are signed using the signature algorithm provided by TCM, to protect the private data such as encryption keys, and ensure data integrity, confidentiality, and security of the data through the transmission of the trusted chain. Identity authentication and encryption key mechanisms ensure the credibility of the computing environment.

**Figure 6:**
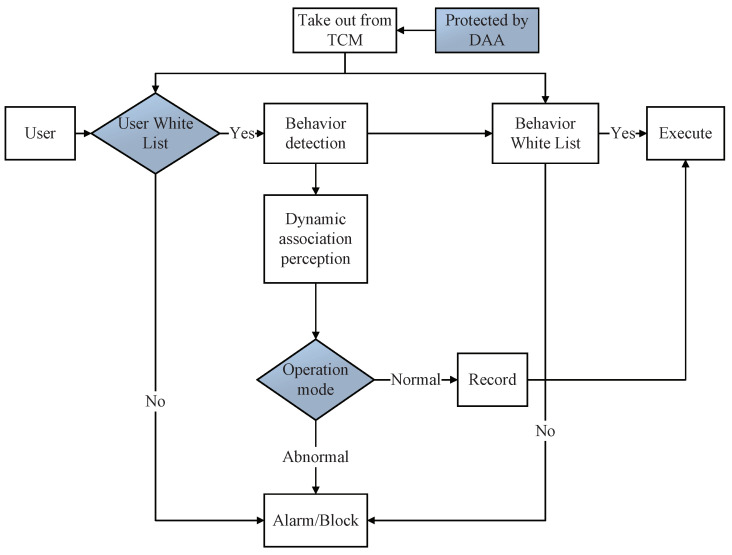
Trusted computing remote attestation and whitelisting.

Whitelist is adopted for application identification, and protection, such as NFD, PSYNC; only a few trusted applications are allowed to run in the current network system to realize the supervision and protection of the whole life cycle of applications from startup, loading, and operation, which reduces the load of the system and improves the availability of the system, as shown in Figure 6. The application developers use their private key to issue the application signatures and register them with the MIS, or the MIS trusted service to provide the verification benchmark and establish the application white list database. For applications on the whitelist, we conduct essential behavior analysis on their regular operations, and establish the whitelist behavior rule base of the applications. When an application starts, the hash measurement value of the application is obtained and compared with the expected benchmark value taken from the trusted module (TCM) to complete the integrity measurement. The execution of the application will be allowed only when the application is integrity and has not been tampered with. At the same time, during the operation of the application, the running status of its key behaviors is monitored in real-time, and the pre-established application behavior rule base is compared to detect abnormal behaviors in time, and record their operations in the log, to trade off between security and availability, and ensure the orderly operation of the application.

At the same time, the security of TCMs needs to be guaranteed. [35] The feasibility of trusted computing storage and computing environment can be confirmed remotely through remote attestation Direct Anonymous Attestation (DAA) technology [14]. DAA uses the Carmenisch–Lysyanskaya signature mechanism [36] to sign certificates of the public key generated by the TCM to ensure the legitimacy of the TCM module. The TCM module uses the DAA certificate to interact with the DAA verifier, thereby identifying and distinguishing the compromised TCM module and ensuring the security of the trusted root.

## 4. Dynamic Defense

Static defense methods, such as identity authentication, data signatures, blockchain, and trusted computing, provide sufficient external barriers for network security to obtain balanced network confidentiality, integrity, and availability and hence protecting network nodes’ security. They can effectively prevent and trace security problems. At the same time, nodes in the network are not simply isolated. They may face group network problems and new types of severe or unknown attacks. Therefore, it is necessary to build an effective dynamic defense system. The measures taken into consideration by DDESS include situation awareness and network security vaccine training and distribution.

### 4.1. Situation Awareness

Network situation awareness refers to the acquisition, understanding, and display of security elements that can bring about the network situation changes in a large-scale network environment, as well as the prediction of network development trends [37,38]. It is divided into network security situational extraction, understanding, and prediction.

When a network individual conducts a static defense, numerous original log files and backbone network traffic data will be generated, including user usage logs and alarm information, which provides a wealth of training materials for dynamic defense situation awareness of network security.

DDESS builds a decision tree containing all or part of the rules in the security policy rule set on the log file, which parses, filters, omissions, and normalizes the entries in the log file. Firstly, the raw data generated by the network monitoring equipment and management system are preprocessed, including data cleaning, noise reduction, dimensionality reduction, standardized merging or conversion, and data verification, removing duplicate and redundant information, merging similar information, and correcting error information, in order to obtain standardized asset data sets, threat data sets, and vulnerability data sets. Then, it carries out data correlation analysis and uses expert knowledge to model network activities and their regulations and characteristics, and identifies the existence and forms of various individuals in the network, and further identifies three different behaviors: malicious attack behavior, abnormal risk behavior (including weak password, account risk login, remote control), and normal access behavior. Behaviors that do not conform to the behavior baseline will trigger behavior alarms to detect risks in time and better find hidden attacks.

Based on the identified attack activities and their characteristics, the attacker’s intention is inferred by further analyzing the semantics of these attack activities and the possible correlation among them. Its main tasks include identifying the source and type of these attack activities and judging the attacker’s capability, opportunity, and the possibility of conducting a successful attack. Based on the network attack chain, network traffic abnormalities are found, such as external attacks, internal malicious scanning, ARP spoofing attack, and internal illegal accesses. The attacker’s intentions can be mainly analyzed from the attack’s behavior and target. The attack behavior prediction analyzes the logical relationship between attack behaviors and infers possible changes. In addition, we need to consider the function and importance of network assets to infer the attacker’s attack intention and source. DDESS situational understanding draws portraits of user behaviors, including individuals and groups. Moreover, it constructs the network attack knowledge graph and draws the security graph from different perspectives, such as external attacks, internal horizontal penetration, and network data leakage. Therefore, DDESS can understand the current overall network security situation, detect and discover security events, and analyze and evaluate the network vulnerabilities and the impact of attacks.

DDESS first checks the network’s security status to achieve network security situation awareness. Then, it understands in detail the various assets and participants within the networks and their potential vulnerabilities to attacks. It finally draws a graph of the network assets and the corresponding security vulnerabilities. After comprehensively acquiring network threat status data, convolutional neural networks (CNN) [39] are used to evaluate the potential network security risks and the behavioral patterns of different nodes. The resulting infliction of existing attack behaviors is based on the current network status and the identified attack activities, along with the vulnerabilities of network assets, as shown in Algorithm 1.
**Algorithm 1** Training of security situational project model**Input:**  The total number of layers *L*, the number of neurons in each hidden layer and output layer,  activation function *f*, loss function, iteration step η,  maximum iteration times MAX and stop iteration threshold ϵ**Output:**  linear relation coefficient matrix ω and bias vector *b* of each hidden layer and output layer.1: Input the risk vector dataset $R=\left\{r_{1},r_{2},r_{3},\cdots ,r_{n}\right\}$.2: **for** m=1 to L **do**3:  Extract the m− layer features $\delta^{m}$ with a convolution kernel     $\delta^{m}\left(r\right)\leftarrow f\left(\sum \omega^{m}\delta^{m-1}+b_{m}\right)$4:  Reduce network scale with max pooling:      $\delta^{m}\left(r\right)=maxpooling\left(o^{m-1}\left(r\right)+b_{m}\right)$5: **end for**6: update the weight ω with backpropagation:7: **for** l=L to 2 **do**8:  Compute $\delta^{l}$ based on $\delta^{l+1}$ and $\omega^{l+1}$ and $z^{l}$9:  Compute the gradient $▽\omega^{l+1}$ and $▽b^{l}$10:   Update $\omega^{l}$ and $b^{l}$ in *l*th layer:    $\omega^{l}\leftarrow \omega^{l}-\eta \sum_{i=1}^{m}\delta^{i,l}{\left(a^{i,l-1}\right)}^{T}$   $b^{l}\leftarrow b^{l}-\eta \sum_{i=1}^{m}\delta^{i,l}$11:   **if** Δω<ϵ or L>MAX **then**12:    break;13:   **end if**14: **end for**

The overall time complexity of Algorithm 1 is the cumulative time complexity of all convolution layers

(1) $$\;\mathrm{Time}\;:O\left(\sum_{l=1}^{D}m_{l}^{2}\cdot f_{l}^{2}\cdot C_{l-1}\cdot C_{l}\right),$$

and the spatial complexity is

(2) $$\;\mathrm{Space}\;:O\left(\sum_{l=1}^{D}f_{l}^{2}\cdot C_{l-1}\cdot C_{l}+\sum_{l=1}^{D}m_{l}^{2}\cdot C_{l}\right)$$

where *D* is the network depth, $f_{l}$ is the filter size, $C_{l}$ is the filter number and the output channels number of layer *l*, and $C_{l-1}$ also represents the input channels number of layer *l*. $m_{l}$ is the size of the output feature map, and $m_{l}=\left\lfloor \left(n_{l}-f_{l}+2*p_{l}\right)/s_{l}\right\rfloor +1$, where $n_{l}$ is the size of the input matrix, $p_{l}$ is the padding, and $s_{l}$ is the stride. In addition, we eliminate gradient vanishing problem with batch normalization [40] and introduce max pooling layer and 1×1 convolution kernel [41] for dimensionality reduction to reduce both the time and space complexity. DDESS can predict the future security status and the changing trend of the network and takes effective defensive measures.

### 4.2. Network Security Vaccine Training and Herd Immunity

The active immune trusted computing technology adopted in static defense can provide immune capabilities for network information systems. The vaccine culture used in organisms usually selects pathogens with strong immunogenicity, loses toxicity through biochemical inactivation or repeatedly immune iteration of animal cells, and retains its immunogenicity.

Biological vaccines are usually injected into organisms to produce immunity. However, network vaccines are different from biological vaccines in a way that they aim to provide a real-time and automatic defense. While defending against attacks, such vaccines improve the network’s resilience and elasticity to maintain its availability and thus return to a normal state eventually. Therefore, the cultivation of network defense vaccines needs to be carried out dynamically in the network environment. The network vaccine can be described as:

(3) V:(antigen,srcaddr,destaddr,timestamp,protocol).

The vaccine of network security’s immunity is a string extracted from the characteristics of data packets transmitted over the network. Antibodies are the measures taken in static defenses and the rules generated in dynamic defenses. During the matching process of antigens and antibodies, the immune cells constantly defend against network attacks, form clonal regeneration, and evolve into memory cells. The memory cells record accurately the network intrusions that have occurred, and encapsulate them into a vaccine cell after stamping them with a timestamp according to the vaccine format as shown in Equation (3).

The encapsulated network security vaccine is transmitted to the vaccine defense center of the adjacent network to be finally distributed among the network nodes. The selection of such a network considers the local routing table, routing, and forwarding. When the antigenic match with the vaccine happens, it becomes activated and can detect network attacks. At the same time, network nodes can also be used for training and cultivating network vaccines as described by Equation (3). When new types of attacks are discovered, these nodes will continue training and learning to enhance the vitality of antibodies in vaccine cells to improve the autoimmunity and achieve adversarial collaboration.

## 5. Experiments

We evaluated the overall defense effectiveness, and intrusion detection performance of DDESS compared with related state-of-the-art schemes in this section.

### 5.1. Overall Performance Evaluation

**Figure 7:**
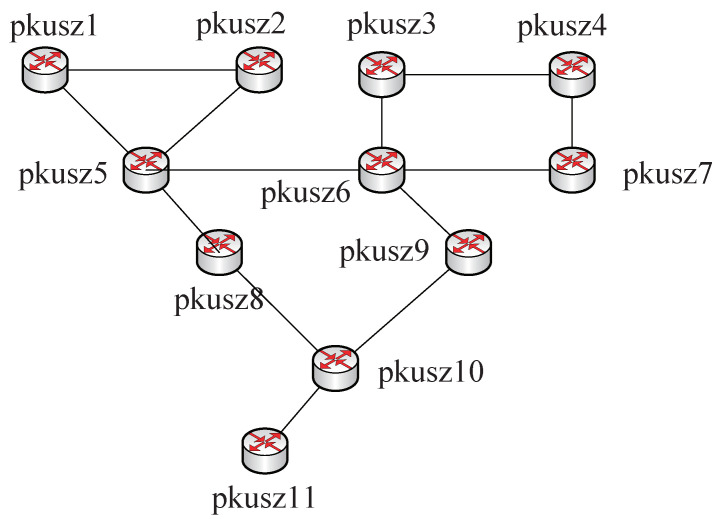
DDESS performance evaluation topology.

Experiments were conducted on a testbed using the topology presented in Figure 7. The router pkusz11 plays the role of the edge router that is connected to the Internet. Experiments and simulations are based on the published KDD-99 dataset that consists of 5 million records [42]. These records are totally made up of 41 attributes and one attack category field which marks all observations as either “normal” or “attacked” with one of the following attacks: Denial of Service (DoS), Remote to Local (R2L), User to Root (U2R), and Probing/surveillance. We carry out attack experiments on DDESS combined with the multi-identifier network. The selected attack methods are conventional under IP networks, such as target detection, attack injection, ARP attacks, and so on. Experiments’ results are demonstrated in Table 1, where ✓ indicates that the attack was successful, while ✗ represents an attack failure.

MIN can effectively defend against target detection attacks such as host discovery, ping scanning, and operating system recognition. It can also prevent Trojan attack injections and one-sentence shell attacks. Attacks within the action phase were divided into ARP disconnection and ARP spoofing. We used “arpspoof” to send fake MAC-IP binding packets, hence poisoning the gateway’s ARP cache. The attacker can therefore disconnect the target network or monitor its traffic. For the ARP disconnection attack, ARP spoofing was first initiated with an arpspoof tool to change the IP forwarding path from one target host to another within the LAN. Both types of ARP attacks were successfully blocked in MIN as shown in Table 1.

### 5.2. Performance of Dynamic Defense

**Figure 8:**
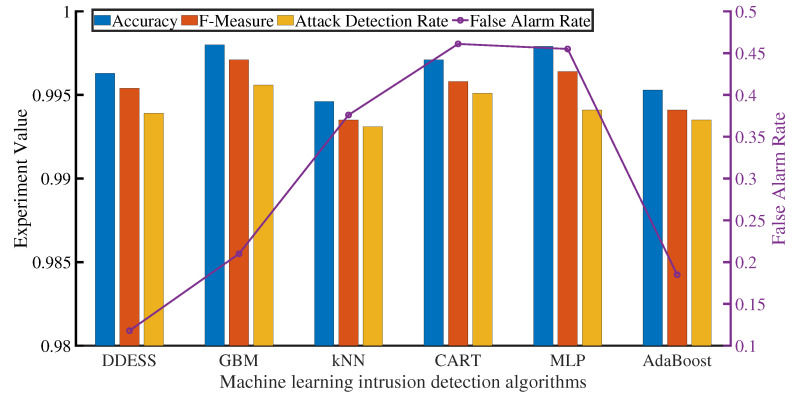
Performance comparison of intrusion detection.

We compared the intrusion detection performance of DDESS on the KDD-99 dataset [42] with that of state-of-the-art machine learning based methods with 10-fold cross-validation. Our comparison references included Gradient Boosted Machine (GBM) [43], k-Nearest Neighbor (kNN) [44], Classification and Regression Trees (CART) [45], Multi-Layer Perceptron (MLP) [46], and AdaBoost [47]. The evaluation results are depicted in Figure 8.

It was observed that, compared with other algorithms, GBM, MLP, and CHAT have better performance in accuracy, reaching 99.81%, 99.79%, and 99.71%, respectively. In order to trade-off the precision rate and recall rate, F-Measure was usually applied [48],

(4) $$F\text{-}\mathrm{Measure}=\frac{(1+\beta^{2})P\times R}{\beta^{2}P+R},$$

where *P* means the precision rate and *R* represents the recall rate. Here, we chose $\beta^{2}=1$ because we attached importance of both *P* and *R*. GBM still achieved the best F-Measure value (99.71%) across all the algorithms, whereas kNN obtains the worst one (99.35%).

As far as attack detection rate (ADR) is concerned, algorithms which are adaptive to continuous attacks have better effects. In Figure 8, GBM (99.56%) and CART (99.51%) can outperform other methods in ADR.

In addition, DDESS can acquire the best false alarm rate (0.118%) because the deep neural networks can reduce the opportunities for false positives. A low false alarm rate and low latency are important for time-sensitive networks (TSN) of Internet of Things (IoT) [31]. Therefore, in order to better monitor network security, we proposed DDESS to comprehensively measure these indicators and come to trade-offs.

**Figure 9:**
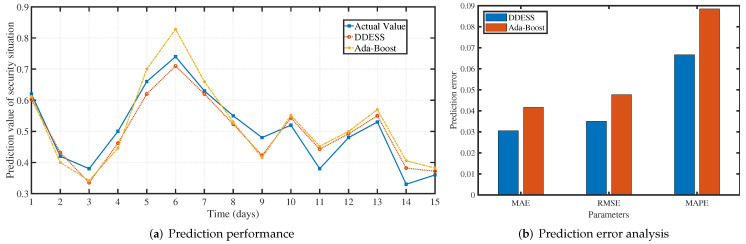
Prediction performance comparison on situation awareness.

We further evaluated the prediction performance of dynamic defense and compared it with TSA-AdaBoost [49], a situation awareness algorithm based on the AdaBoost machine learning method. DDESS adopts the batch normalization [40] to reduce the influence of gradient vanishing problem, and max-pooling layer and 1×1 convolutional kernel [41] for dimensionality reduction. Therefore, the overall fitness of the proposed DDESS is better than TSA-AdaBoost for the prediction of test samples, as shown in Figure 9a. We select mean absolute error (MAE), root mean square error (RMSE), and mean absolute percentage error (MAPE) as the prediction evaluation metrics to evaluate the proposed prediction model, where

(5) $$\mathrm{MAE}=\frac{1}{N}\sum_{i=1}^{N}\left|\hat{y_{i}}-y_{i}\right|$$

(6) $$\mathrm{RMSE}=\sqrt{\frac{1}{N}\sum_{i=1}^{N}{\left(\hat{y_{i}}-y_{i}\right)}^{2}}$$

(7) $$\mathrm{MAPE}=\frac{100\%}{N}\sum_{i=1}^{N}\left|\frac{\hat{y_{i}}-y_{i}}{y_{i}}\right|,$$

where $\hat{y_{i}}$ means the prediction situation value. As shown in Figure 9b, the MAE and RMSE values of DDESS are 0.0306 and 0.035, respectively, while these of TSA-AdaBoost are 0.0417 and 0.0486, respectively. Compared with TSA AdaBoost, DDESS has less overall error precision in predicting network security situation value. In addition, the MAPE value of DDESS and TSA-AdaBoost is 6.67% and 8.95%, respectively, which means that DDESS has better accuracy than TSA AdaBoost.

Let us analyze forecast results in Figure 9a from a network administrators’ perspective. Although the situational value on day 3 is relatively low, it is predicted that the network situational value will have a higher tendency on the next day (i.e., day 4), which will warn network administrators about the occurrence of network attacks. At the same time, based on the 11th-day forecast trend, this is likely to be the end phase of network attacks, which will make administrators pay special attention to the network behavior logs for the next two days to ensure that they are not deleted or destroyed by attackers. On the other hand, the forecasts on the 12th and 13th show that network attacks continue, and some vulnerabilities probably exist in the network system.

DDESS can train the attack model and make corresponding portraits of the attack and the attacker without requiring additional hardware, such as FPGA. To perceive the motivation behind the attack, DDESS will classify and mark the attack behaviors of the same source. This helps learn and predict potential attacks behaviors from the same source in the future.

### 5.3. Competition and Trial

The multi-identifier network system (MIN) combined with DDESS technology has withstood the “Network Security Challenge Competition” held by the Purple Mountain Laboratory [50]. Forty-eight teams continuously carried out remote online high-intensity network attacks for 72 h. During this period, the cumulative number of attacks reached 3.58 million. More precisely, the MIN was one of the few network security systems any team has not broken through. It also successfully prevented most competition-related qualification attacks such as Linux privilege escalation, virtual machine escape, session forging and hijacking, and simulation PWN. The DDESS-based system has been proved to be more robust when compared with traditional commercial workarounds.

## 6. Conclusions and Future Work

To overcome the current internet security problems, we propose a double defense strategy with endogenous safety and security (DDESS) based on the MIN system. DDESS provides both static and dynamic defense strategies against different network attacks. The proposed system preserves network security using static defensive measures such as identity verification, encryption protection, and trusted computing. It also uses dynamic defensive measures like situation awareness and vaccine cultivation and distribution. Experiments and performance analysis showed that DDESS provides sufficient availability and robust security compared to other existing network defense solutions. In the future work, we plan to introduce edge computing technology to reduce the pressure of central servers and better adapt to the network development, such as 6G, Internet of things (IoT), etc. In addition, we will study the intrusion detection algorithms, and put forward more effective strategies for network security detection and prediction in our future work.

## Figures and Tables

**Table 1:** Attack results of experiments.

Attack Phase	Description		IP-IP	IP-MIN
Target detection	Host discovery		✓	✗
	ping scan		✓	✗
	OS recognition		System fingerprints obtained	Host non-survival
	Port scan		All ports probed	The host is alive, but no port detected
Attack injection	Trojan	TCP trojan	✓	✗
		UDP trojan	✓	✗
		ICMP trojan	✓	✗
	One-sentence shell		✓	✗
Action	ARP Attack		Information sniffing	Target cannot be sniffed
			Network disconnection attack	Target not affected

## Data Availability

The data used for this research is publicly available at https://kdd.org/kdd-cup/view/kdd-cup-1999/Data.

## Abbreviations

The following abbreviations are used in this manuscript:

DDESS	Double Defense strategy with Endogenous Safety and Security
NDN	Named Data Networking
MIN	Multi-Identifier Network
MIS	Multi-Identifier System
MIR	Multi-Identifier Router
PoV	Proof of Vote
DAA	Direct Anonymous Attestation
TCM	Trusted Computing Module
ACC	Accuracy
ADR	Attack Detection Rate
FAR	False Alarm Rate
